# Interplay between Tradition and Modernity: Stress and Coping Experiences among Parents of Children with Autism in Beijing, China

**DOI:** 10.3390/bs13100814

**Published:** 2023-10-02

**Authors:** Xiaoran Wang, Fuhua Zhai, Yixuan Wang

**Affiliations:** 1 Faculty of Humanities and Social Sciences, Beijing University of Technology, Beijing 100124, China; 2 Beijing Social Governance Research Center, Beijing 100124, China; 3 Graduate School of Social Service, Fordham University, New York, NY 10023, USA; fzhai1@fordham.edu; 4 Department of Social Work, China Youth University of Political Studies, Beijing 100089, China; ywang134@fordham.edu

**Keywords:** children with autism, China, Chinese culture, family support, parenting

## Abstract

In traditional Chinese culture, specific beliefs and values can influence parents’ experiences of stress and coping while raising children with autism. However, as China undergoes rapid social changes amid globalization, are these cultural influences still significant for today’s parents of young children with autism? This study delves into this question through 12 in-depth interviews with parents of children with autism in Beijing. Content analysis indicated that while influences from traditional culture remain, modern parents often diverge from them. They adopt Western views on autism to mitigate stigma, establish boundaries with grandparents to ensure effective early interventions, address imbalanced professional dynamics, adjust authoritarian parenting styles, and broaden their social networks. A mix of traditional and contemporary parenting characterizes their experiences. The discussion elaborates on the findings, emphasizing the importance of family support.

## 1. Introduction

How parents perceive and respond to stress while caring for a child with autism is a socially constructed process [1,2]. While the existing literature has extensively documented the profound influence of traditional Chinese culture on parents’ stress and coping experiences when raising children with autism [3,4], there is a pressing need to revisit this topic. In the context of globalization, Chinese parents are increasingly exposed to Western perspectives on autism and corresponding interventions [5]. This exposure is expected to bring about shifts in their reactions upon receiving such a diagnosis. Furthermore, Chinese parents born in the 1980s are raising their children in a society marked by evolving social policies and the expansion of social services [6]. Consequently, they are likely to have more support mechanisms in place to manage parenting stress than previous generations. Added to this, as education levels rise in China [7], the parents of young children with autism may now be better positioned to challenge traditional Chinese cultural norms.

The study aimed to explore two research questions: (1) How does traditional Chinese culture continue to influence the stress and coping experiences of today’s parents of children with autism? (2) How do their experiences reflect the evolving culture of China? We expect that the parents of young children today, while still impacted by traditional cultural norms, will exhibit deviations from them. Their experiences are characterized by the interplay between persistent conventions and shifting dynamics.

## 2. Literature Review

To provide a solid foundation for the study, a comprehensive literature review was undertaken from several dimensions, covering the general influence of culture on stress and coping, notable effects of culture on stress and coping when parenting children with autism, and the distinct influence of Chinese culture on parental experiences across mainland China, Hong Kong, Taiwan, and Chinese communities in Western countries. Furthermore, studies addressing recent changes that may affect parents of children with autism in China were explored to highlight the importance of revisiting this topic.

### 2.1. Influence of Culture on Stress and Coping

Culture has been defined as “a set of behavioral norms, meanings, and values or reference points utilized by members of a particular society to construct their unique view of the world and ascertain their identity” [8]. One of the primary ways culture influences stress and coping is by shaping how people perceive and respond to events. According to the foundational Transactional Model by Lazarus and Folkman (1984), individuals perceive a situation as stressful if they view it as a threat to their interests (primary appraisal). They then choose coping strategies based on their available resources (secondary appraisal) [9]. Cultural beliefs and values can influence these perceptions. Taking the well-known dichotomy of individualistic and collectivist cultures as an example, people in individualistic cultures often tie events to personal success or failure, leading them to a problem-oriented approach that relies on individual efforts. In contrast, those in collectivist cultures link whether an event is stressful to the group’s success or failure, and they tend to lean toward emotion-oriented strategies, drawing strength from the collective [2].

Culture also influences stress and coping by forming social norms. For instance, in Asian cultures, both immediate and extended family systems play a central role in an individual’s coping mechanisms during stress due to communal norms [10]. There is a tendency among individuals in these cultures to avoid seeking professional help, stemming from a reluctance to disclose personal stressors and worries about the implications for their relationships [11]. This contrasts with some Western cultures where there is a greater emphasis on independence and seeking support from broader social networks or professionals [12]. For another example, gender roles, deeply entrenched in cultural norms, affect how individuals perceive and cope with stress. In some cultures, women bear the brunt of responsibility for the emotional well-being of family members and frequently shoulder significant caregiving duties [13], while men are often expected to adhere to norms of emotional restraint and stoicism [14]. Although a more extensive elaboration is constrained in the limited space, it is evident that any experiences related to stress and coping should be examined within a cultural context [2]. 

### 2.2. Influence of Culture on Stress and Coping in Parenting Children with Autism

In the research field of parenting children with autism, the roles of culture in parents’ stress and coping have revolved around the following aspects. First and foremost, autism is understood differently across cultures. In many Western societies, such as the United States, autism has been reconceptualized as a neurological condition rather than a disability, emphasizing differences rather than deficiencies, particularly following disability rights movements [15]. However, in some non-Western contexts, autism is still perceived as a mental illness, resulting in a social stigma that can induce feelings of shame among family members [16]. In particular, this stigma surrounding autism can also be linked to alternative explanations of the condition, even in the face of medical evidence. For instance, in Korean culture, autism is sometimes mistaken for Reactive Attachment Disorder (RAD), placing considerable blame and guilt on parents, especially mothers [17].

Second, alongside factors such as a child’s autism severity and financial hardship, culture plays a pivotal role in shaping parents’ stress appraisal and coping behaviors [18,19]. While high levels of parenting stress among parents of children with autism are evident across countries [20], nuanced differences in parenting stress have been observed between cultures. A comparative study between parents in Japan and the United States showed that parents from collectivist cultures often feel more discomfort due to their autistic children’s differences and are more sensitive to criticism about lax discipline or unfulfilled parental duties [21]. Another study highlighted that owing to high expectations for their child’s academic success, parents within Asian cultures often experience greater parenting stress than their non-Asian counterparts [22]. With regard to coping styles, cross-cultural differences are prominent. Research indicates that on average, Asian parents of children with ASD utilize problem-focused coping strategies more frequently than Caucasian parents [19,23,24]. However, some cultural concerns like “saving face” often discourage Asian parents from help-seeking and instead lead them toward avoidance coping strategies [19,24].

### 2.3. Culture-Related Parenting Stress among Chinese Parents of Children with Autism

In the specific context of Chinese culture, the stress experienced by parents raising children with autism is closely intertwined with cultural factors, especially during the crucial diagnosis phase. Rooted in traditional etiological beliefs, some parents perceive their child’s autism as a consequence of past transgressions or ancestral sins. This perspective often leads to an internalization of guilt and self-blame [25]. Complicating matters further is the language used to describe autism in Chinese culture, which includes terms such as “gu-du zheng” and “zi-bi zheng”, which are translated as “being self-closed” or “being lonely”, obscuring the medical essence of autism [26]. This dual interpretation not only evokes conventional reactions such as shock but also represents a cultural misunderstanding [27].

The stigma associated with disabilities is notably amplified within Chinese culture, often manifesting through the term “canfei”, which carries connotations of worthlessness [25]. Such perceptions significantly contribute to the devaluation of children with autism and make parents vulnerable to criticism, fostering feelings of shame and adversely affecting parental mental well-being [28]. In the context of family dynamics, where the paramount importance of family honor is ingrained, having a child with autism can be perceived as tarnishing the family’s reputation, which is a notion deeply rooted in cultural attitudes [29]. These beliefs, underpinned by the interplay of shame culture and familism, ultimately contribute to elevated parental stress.

The cultural context of Chinese parenting norms adds another layer of complexity. These norms are built upon the longstanding expectation of filial piety, wherein children are traditionally expected to care for their elders in their adult years [30]. However, when a child with a disability is born, these conventional parenting expectations are disrupted. This can result in family conflicts and, in some cases, separations, further burdening mothers, as they primarily shoulder the caregiving responsibilities [31,32].

### 2.4. Culture-Related Coping Experiences among Chinese Parents of Children with Autism

Cultural influences continue to play a substantial role in parents’ coping strategies when faced with the challenges of raising children with autism. A predominant approach among Chinese parents is avoidance, which is driven by cultural motivations such as safeguarding the family’s honor [33]. While the practice of concealing children with autism from society is not unique to China, its prevalence is augmented by the cultural importance of “face” or “mianzi” [33]. Furthermore, despite the evident need for professional assistance, some Chinese parents, including those residing in Western countries, exhibit a reluctance to seek such help [4,34]. This hesitancy often stems from cultural norms that emphasize deference to authority and the value of interpersonal harmony, discouraging open disagreement with professionals [34,35,36].

Alternatively, parents frequently resort to problem-focused coping mechanisms to address the needs of their children with autism, particularly in domains such as childcare, early interventions, and special education. The cultural expectation that parents will play the primary role in their child’s education requires them to assume multifaceted responsibilities within the household, where they serve as caregivers, educators, and trainers [37,38]. Within a collectivist culture that places a high value on social responsibilities, personal obligations, and family unity, familial ties often grow stronger. Grandparents often contribute significantly to daily parenting activities [39], and parents demonstrate remarkable self-sacrifice by altering their personal lives for the well-being of their children [32,40,41]. However, differing viewpoints among family members can, at times, lead to heightened interpersonal stress within intergenerational caregiving dynamics, and parents’ tendency to overlook their own needs can further contribute to heightened stress levels [40].

### 2.5. Recent Shifts in Chinese Culture Related to Autism

Previous research has shed light on how traditional Chinese culture shapes the stress and coping experiences of parents raising children with autism. However, the question of whether these dynamics have evolved remains open, warranting further investigation for several reasons.

First and foremost, China is making rapid strides in the social inclusion of children with autism and their families. At the national policy level, the State Council of China revised the Regulation on the Education of Disabled Persons (first issued in 1994) and issued the Regulation on the Prevention and Rehabilitation of Disabled Persons in 2017. The former strives for inclusive education in mainstream schools and preschools, while the latter promotes rehabilitation services, encompassing improved facilities, medical insurance coverage, and community support [6]. Additionally, China’s Disabled Persons’ Federation (CDPF), a national organization representing individuals with disabilities, initiated public autism education in 2015. Annually, on 2 April, which is World Autism Awareness Day, diverse local events are organized across China [42,43]. Furthermore, an increasing number of self-help organizations, whether initiated by individuals or sponsored by the government, are advocating for policy shifts, notably inclusive education for children with intellectual disabilities [44]. These transformative efforts foster optimism that the traditional cultural stigma linked to autism in Chinese society may diminish.

Secondly, through the process of reform and opening up since the 1980s, China has progressively engaged with Western cultures and perspectives, potentially influencing how parents navigate the challenges of raising children with autism. Studies have indicated that Chinese immigrant parents residing in Western societies are more inclined to attribute autism to genetic causes [40], seek early intervention services [45], and actively advocate for their children within the education and social service systems [36]. However, the extent to which local Chinese parents exhibit similar tendencies when exposed to Western cultural influences remains underexplored. Additionally, Chinese parenting practices have gradually incorporated certain Western characteristics. The newer generation of Chinese parents demonstrates heightened concerns for their children’s emotional well-being and social connections while adopting less authoritarian parenting styles, such as control and shaming [46]. Nevertheless, the impact of these shifts on how parents raise children with autism remains relatively unknown.

Thirdly, as of 2021, China’s working-age population boasts an average education level of 10.9 years, which is equivalent to the second year of high school. Notably, 24.9% of them have higher education qualifications, marking a significant increase of 10.3 percentage points since 2012 [7]. Previous research has established the substantial impact of education on individuals’ health-related behaviors [47] and their utilization of mental health services [48]. For instance, a study revealed that among Chinese individuals with schizophrenia, those with a high school education or above were more likely to engage in psychological counseling and exhibit greater tolerance toward others but also tended to conceal their diagnosis from others [49]. These findings suggest that education might potentially enable parents of children with autism to counterbalance or reinforce the influence of traditional culture on their parenting experiences.

For these reasons, this study undertook the task of investigating the parenting stress and coping experiences of Chinese parents raising children with autism from a cultural standpoint. Within the rapidly evolving landscape of China, the study’s objective was twofold: to identify enduring traditional cultural perceptions that continue to impact the population and to unravel emerging cultural tendencies.

## 3. Method

### 3.1. Participants and Recruitment

This study used a qualitative research approach, seeking in-depth insights into participants’ experiences through one-on-one interviews [50]. A purposive sampling strategy was employed to deliberately recruit respondents based on their potential to provide the requisite information [51]. Parents who met all the inclusion criteria were eligible for this study: (1) having a child with diagnosed or suspected autism; (2) self-identified as the primary caregiver of a child with autism; and (3) seeking or once having sought early intervention services in Beijing in the past 3 years. Parents of young children with autism were the focus of this study because their experiences best reflect the influence of culture on family-based early childhood care, which was a stage at which family support services are much needed.

For recruitment, the primary researcher established a strong rapport with three local early intervention and special education service providers in Beijing, including a manager of an inclusive preschool, a manager of an art studio for children with autism, and a university professor in psychology who served as a clinician for children with autism. Two of these providers distributed flyers to their clients and referred parents who were interested in participating in the study, resulting in the recruitment of three parents. One provider invited the researcher to deliver a presentation on self-care for parents of children with autism in a small parenting group, facilitating the on-site recruitment of two parents. Additionally, seven more parents voluntarily joined the study through snowballing referrals within the parents’ network. Table 1 shows the characteristics of all 12 participants. The sample was predominantly composed of mothers (nine participants) and parents with college-level education or above (nine parents).

### 3.2. Data Collection

One-on-one in-depth interviews were conducted in Mandarin Chinese from August to October 2019 either in person or via phone depending on the participants’ preferences. A semi-structured interview guide was developed, including questions about parents’ experiences related to the diagnosis of autism, the utilization of early intervention services, parenting practices, and parents’ self-reflections on how Chinese culture influenced these experiences. The interview guide is included in Appendix A. On average, each interview lasted approximately 2 h. All participants provided consent for audio recording. Ethical approval for the research was obtained from the Institutional Review Board (IRB) at Fordham University, the institute with which the primary researcher was affiliated when the study was conducted.

### 3.3. Data Analysis

In this study, we adopted a content analysis approach for data analysis, recognizing it as a systematic technique used to identify trends and patterns in textual data while striving for objectivity [52]. The objectives of content analysis, which include analyzing communication content trends and making inferences about aspects of culture and cultural change [53], guided our analysis. Specifically, we combined deductive and inductive content analysis approaches to identify the stress and coping experiences of study participants that aligned with traditional culture and those representing emergent characteristics. The analysis included three stages: deductive analysis, inductive analysis, and synthesis. The coding process details are presented in Table A1, which is provided in Appendix B.

#### 3.3.1. Deductive Content Analysis

The primary objective of this stage is to assess the applicability of earlier theories or knowledge to the current context [54]. Given that previous research has explored the influence of Chinese culture on the parenting experiences of Chinese parents of children with autism and yielded similar results, the existing knowledge served as a foundational basis to investigate whether today’s parents of young children continued to adhere to traditional cultural norms. Based on the existing literature, the first author drafted a categorization matrix comprising codes related to the influence of Chinese culture on parents’ stress and coping experiences. Simultaneously, the second author reviewed the literature and engaged in deliberations with the first author to refine the matrix, culminating in a consensus. The final matrix encompassed 18 codes. Subsequently, the first author thoroughly read the interview transcripts, using a line-by-line coding method to assign the predefined codes to the relevant sentences. This process involved multiple iterations of reading and coding to ensure accurate assignment of the codes.

#### 3.3.2. Inductive Content Analysis

This approach is usually conducted to describe human experiences and perspectives when previous knowledge is either lacking or fragmented [55]. In this study, the aim was to uncover new aspects of stress and coping that might suggest a diminished cultural impact. To achieve this, the first author revisited the interview transcripts, specifically seeking any stress and coping narratives not already included in the original matrix. These sentences were assigned open codes, allowing for a more comprehensive exploration of novel aspects of the participants’ experiences. Subsequently, the primary author compared the similarities and differences across the open codes and grouped them into 11 sub-concepts (i.e., Western perspectives on autism, generational attitudes toward autism and early intervention, family boundary tensions, awareness of rights, collaboration initiatives, ”renqing” investment to cultivate professional relationships, balancing discipline and freedom, stress from competitive parenting, stress from urban educational culture, biased media portrayals, and expanding social circles). To ensure the credibility of the coding process, the third author, serving as a peer examiner, conducted a thorough review of the transcripts and codes, providing feedback and engaging in discussions to assess the reliability and validity of the assigned codes.

#### 3.3.3. Synthesis

The research team engaged in in-depth discussions regarding the relationship between the initial matrix and the sub-concepts derived from the open codes. While the majority of the sub-concepts primarily reflected the influence of Chinese culture, a few sub-concepts stood out, indicating a departure from traditional cultural norms. Finally, the predetermined codes generated in the deductive stage and the sub-concepts derived during the inductive stage were systematically analyzed. This comprehensive analysis led to the identification of five overarching themes that depicted the stress and coping experiences of the study participants, which are shaped by the interplay of traditional conventions and contemporary society. The subsequent section introduces these themes in detail.

## 4. Findings

### 4.1. Blending Tradition and Modernity in Perceptions of Autism

The stigma surrounding mental illness remains a persistent source of stress for Chinese parents of children with autism. During the interviews, two parents directly expressed their concern about the official classification of autism as a mental disability (in the Second National Sample Survey of Disabled Persons in China conducted in 2006, autism was officially classified under the category of mental disabilities [56]. At present, the screening and intervention for children with autism are still officially categorized under the domain of mental health [57]), which left them feeling deeply stigmatized (Participants 5 and 8). To cope with this pressure, some parents have chosen to reframe the meaning of autism within the context of traditional Chinese culture, while others have sought to broaden their understanding by exploring how autism is defined in Western countries. An illustrative account from one mother vividly demonstrates the impact of traditional Chinese culture on the experiences of parents raising children with autism. Her initial reaction to the possibility of an autism diagnosis reflects the common fear and stress prevalent among parents in China, where mental illness is still shrouded in misconceptions and viewed as intimidating.

*The doctor recommended going to a hospital. I asked, “What kind of this hospital?” He replied, “You’ll find out when you get there. It’s a specialist hospital.” … I left and headed straight to the hospital. When I saw that it was a psychiatric hospital, my mind was blown. [I thought] this place is dreadful.*

(Participant 6)

Following her daughter’s autism diagnosis, she experienced despair and even thought of suicide. However, she found solace in the words of a fortune teller from her rural hometown: “*The child was not ordinary. She is a virgin coming from heaven… She will become quite successful, and I will depend on her [in the future]*”. Her coping experience highlights the profound influence of traditional Chinese culture, where the notion of destiny is still deeply ingrained in folk beliefs.

In contrast, some parents have sought to challenge social stigma by familiarizing themselves with Western perspectives on autism. They have adopted terms such as “developmental disabilities” and “neurodiversity” to emphasize that children with autism should not be stigmatized. One mother arrived at the conclusion that having autism should not be a source of shame after reading books and articles about autism in both Chinese and English.

*I think it is simple to describe autism as a [personal] barrier. Why do I wear contact lenses? It’s because my vision isn’t clear. And that can be corrected. While addressing autism is more difficult, there’s no reason to feel ashamed and conceal it from others, is there?*

(Participant 2)

Similarly, another mother (Participant 5) voiced her concern about the classification of autism under mental illness because it could cause parents of typical children to stay away from children with autism. She said:

*“Some parents [of typical children] in my son’s swimming class quickly noticed my son’s difference. When I explained that it was [due to] developmental barriers, they understood and were open to communication.”*

It is worth noting that while some parents have embraced Western perspectives on autism, it does not completely alleviate their confusion regarding the nature of autism. As expressed by one father (Participant 7), he prefers using the term “gudu” (characteristics) rather than “zibi” (syndrome) to describe autism, as it implies a set of characteristics rather than an illness.

*I have observed people who were not very social and tended to be reclusive since my childhood. Unlike those with Down syndrome, they simply had certain traits [that made it challenging for them] to socialize with others.*

(Participant 7)

### 4.2. Generational Tension in Parenting Children with Autism

In terms of family dynamics, only a few parents reported experiencing parenting stress stemming from blame from other family members for having a child with autism. This might be because the parents in our study were all married and had good bonds with the child’s grandparents (including the in-laws). Moreover, the child’s grandparents often played a substantial role in raising the child, reflecting the traditional Chinese practice of multigenerational family parenting. This active involvement and support from grandparents often alleviated the time constraints faced by parents.

*I am quite pleased with [my daughter’s] grandparents. They cooked meals, washed clothes, and took care of her. Grandpa also escorted her to school. Although grandma didn’t provide formal training, she helped her recognize some basic objects using flashcards.*

(Participant 7)

However, some parents reported experiencing family tensions arising from family members, especially from grandparents, who denied their child’s diagnosis of autism and accused the parents of labeling the child. This disagreement made it challenging to reach a consensus between generations on whether to seek early intervention services for the child.

*I feel isolated at home. I’m the only one who cares. His father always disagrees with me, believing there is nothing wrong with the child… Grandma also scolds me, saying that I claim every day that the child is sick, but in fact, it’s you who are sick.*

(Participant 12)

Furthermore, differences in parenting practices between today’s parents of young children and their parents created challenges. Some grandparents tended to indulge their grandchildren’s demands, making it difficult for parents to address their children’s problem behaviors effectively. Parents often found themselves torn between gratitude for the grandparents’ assistance and concerns about hurting their feelings. A mother said that she and her husband eventually chose to send the child’s grandmother back home so that they could create a parenting environment that was conducive to her son’s early intervention.

*Therapists taught me how to raise my son at home. I’d like to follow what they said. [But] the grandparents interfered, and at times, even opposed it. For example, regarding “not spoon-feeding”, they fed him secretly when we were out… so I let my husband talk to them about it. The grandparents were quite upset. After my husband decided not to heed his parents and the grandparents finally left, my husband and I began implementing interventions at home.*

(Participant 3)

### 4.3. Balancing Unequal Professional Relationships

Regarding relationships with professionals, the traditional Chinese cultural values of respect and obedience to authorities, such as early intervention agencies and professionals, continued to hold significant sway. Confronted with unequal power dynamics, parents, including those who were highly educated and held opinions about their child’s early intervention services, often chose to prioritize maintaining harmony by suppressing their own views.

*I feel that the curriculum might be a bit too easy for my child… but I’m not an expert… so I didn’t say anything. After all, the agency has the expertise, and I should respect their approaches.*

(Participant 8)

*[This agency] is fine. If you have something to say, you can talk to the therapists. Nevertheless, regardless of the agency, parents have to be very careful, plan the communication ahead, and avoid making the decision [on behalf of the professionals].*

(Participant 10)

*When I talked to teachers about my opinions on what they should do, I thought over it again and again to avoid harming their self-esteem.*

(Participant 9)

Another approach parents used to cope with the pressure was resorting to “guanxi” (personal relationships) and “renqing” (favors). This often required spending additional money to cultivate relationships on top of the regular costs and fees of the intervention, leading to additional stress in the Chinese cultural context.

*At that time, the private therapist charged about 400 yuan for a home visit. I would treat him to a meal, which was a kind of renqing [favor]. It costs a lot, but what can you do?*

(Participant 2)

*Our expenses for interventions are already quite high. However, every year on Teachers’ Day, I give several hundred yuan to each of my son’s three teachers. Even though they initially decline, they eventually accept it as long as you persist… One of my friends told me that she gives teachers at least 100 yuan, and maybe even up to 1000 yuan for the Spring Festival [Chinese New Year] so that her son is treated very well… This is such pressure!*

(Participant 3)

However, it is worth noting that advocacy as a coping strategy was mentioned only once by a mother, who used the word “fight” (in Chinese), implying conflicts and confrontations (Participant 2). Another exceptional case involved a mother acting as an advocate (Participant 11). Recognizing the absence of professional support for children with special needs in her son’s general preschool, she approached the principal with a suggestion to invite special education teachers to visit the school. As a result of her proactive efforts, special education teachers from an inclusive preschool began visiting her son’s general preschool once a week, offering direct behavior-related instructions and support.

### 4.4. Adjusting Authoritarian Parenting Styles

During the interviews, parents’ experiences shed light on the prevalent traditional Chinese parenting style characterized by authoritarian practices. Most parents emphasized discipline as a means to address their children’s behavioral challenges, using techniques such as behavior reinforcement and occasional punishment in their daily parenting routines. Notably, parents of children with mild autism tended to adopt an authoritative parenting style, which is driven by their aspirations for their child’s successful social integration in the future.

*I became a bit more demanding of him after his diagnosis. I let him learn more at home so that he wouldn’t feel less in society compared to anyone else. I wanted him to acquire the same knowledge as children his age, so he wouldn’t feel inferior when interacting with them later.*

(Participant 8)

However, parents also reflected on their authoritarian approach and sought to make adjustments. Recognizing that excessive demands for behavioral discipline led to parent–child tension and exacerbated their child’s emotional and behavioral issues, parents turned toward nurturing love and care. This adjustment often occurred when parents encountered a conflict between their roles as a nurturer and a trainer. Notably, it was possible that the application of applied behavior analysis (ABA) might have reinforced parents’ authoritarian parenting style.

*I looked back and thought, what have I trained my child into? When I stared at her, she dared not move… what I used to often think about was to fix her problem behaviors… I constantly pointed out her problems and subjected her to non-stop training…*

(Participant 6)

As parents adjusted their parenting style, they appeared to face challenges in striking a balance between discipline and freedom. When parents reduced their emphasis on discipline to address their children’s emotional needs, they expressed concerns that such adjustments might hinder their children’s progress.

*This year, I have noticed that he has become assertive and is able to express his needs clearly and promptly. After school and before bedtime, we spend a few hours together, and I fulfill his requests as he makes them. For some inappropriate demands, I reason with him. In the beginning, he had some emotional reactions, but now slowly, he can listen and understand… I wonder, is it a waste of time when I spend so much time with him like this?*

(Participant 4)

Notably, there were certain patterns in the adjustment of parenting styles during the interviews. Parents who were within one to two years of their child’s autism diagnosis tended to take on the role of trainers in the family and emphasized the importance of discipline. This inclination may be related to their initial concerns during the early intervention stages about whether their child would make progress quickly. However, as time elapsed since the diagnosis, parents were more likely to adapt their parenting styles and lean toward addressing their child’s psychological and emotional needs. Nevertheless, finding the appropriate balance remains a continuous challenge for parents.

### 4.5. Addressing the Modern Challenges of Social Marginalization

Parents expressed that despite increased media coverage of autism in recent years, the portrayal of children with autism tends to be biased. For example, one of the few Chinese movies about children with autism, titled *Ocean Heaven*, in which an adult with severe autism relied on his father for assistance in daily activities, made both parents and the public feel depressed by the grim future of children with severe autism (Participants 2 and 7). Parents also noted that in most media reports, those with autism were either idealized as geniuses or depicted as difficult to discipline and spoiled brats. Due to the lack of public understanding of autism, parents continued to feel stigmatized and marginalized in society. To avoid this, most parents in the study chose to keep their child’s condition a secret, only confiding in their immediate family members. One father (Participant 4) revealed that he had been avoiding community activities. In his words, the reason was “Why let others say something negative [about my child]?”.

An interesting finding was that parents’ sense of marginalization was exacerbated by the increasing emphasis on early development and childhood behaviors, particularly in Beijing. One mother shared that peer pressure haunted her even before her child started preschool, as parents constantly compared their children’s achievements in her neighborhood. Another mother switched her son’s preschool from Beijing to her rural hometown because parents of typical children in Beijing were intolerant of her son’s minor deviant behaviors and expected her to discipline him rigorously.

*Environments exert pressure on you all the time, especially because we have difficult children. Even when children are very young, parents begin [to compete]. While taking a stroll with your child, you may overhear fellow parents discussing the months when their children started teething or became free from diapers. This is in China!*

(Participant 2)

*It would be the same even if I were to switch to another preschool [in Beijing] because urban parents here care too much about their children and can’t tolerate even a little skin wound. In my hometown, parents don’t fuss over so many things.*

(Participant 12)

At the same time, some parents wanted to create a small, nurturing environment that was amiable to their children. In contrast, some parents sought to create a supportive environment for their children by sharing the diagnosis with close friends and organizing small outings where their children could play together. These efforts provided a sense of relief and reinforced the idea that parents of children with autism shared common experiences with other parents. However, it cannot be denied that stepping out of their comfort zone required parents to confront personal challenges such as shyness and social interaction anxiety, as mentioned by two parents.

*In our [autism] circle, we discuss certain behaviors of children, such as the frequent habit of taking off their shoes. However, one of my coworkers told me, “Your child likes doing it; my son does too.”… When I discovered that parents of typical children faced similar behaviors, I began to worry less… Regarding intervention methods, such as reinforcement, punishment, and reward tokens, all of these can also be applied to typical children…So, parents don’t have to be confined to the autism circle.*

(Participant 5)

## 5. Discussion

Based on the research findings, this discussion focuses on understanding the lasting impact of traditional Chinese culture on parenting children with autism and emerging trends. The interplay between traditional and modern perspectives becomes evident when we examine beliefs about the causes of autism, intergenerational parenting experiences, relationships with professionals, adjustments in parenting styles, and social integration. While traditional Chinese culture continues to play a significant role in how parents approach caregiving, the evolving landscape introduces changes that add complexity and diversity to the experiences of Chinese parents raising children with autism.

### 5.1. Perceptions of Autism

Chinese cultural perspectives on mental illness profoundly influence parents’ understanding of autism, which is consistent with prior research [25]. One mother’s reliance on a fortune teller’s insights vividly illustrates this, echoing old beliefs that link disabilities to past deeds or family history [3]. However, a notable shift was observed among the other parents. Drawing more from Western ideas, they interpreted autism through a blend of traditional and contemporary perspectives. By using terms such as “developmental disabilities” and “neurodiversity,” they can counteract social prejudices. Their education might have contributed to this shift since previous studies have discovered that education level could be related to the understanding of autism [58], although contradicting evidence also exists [59]. Meanwhile, this change could indicate the growing influence of Western perspectives, similar to the experience of Chinese immigrant parents who navigate a mixture of Eastern and Western views on autism [45].

Nevertheless, ambiguity remains, as illustrated by a father who distinguished between “characteristics” and “illness” when discussing autism. Given the Western tendency to perceive autism as a variation in brain development [15], promoting such views in China could transform societal attitudes toward autism [60], emphasizing the potential of children with autism [61]. However, a significant challenge lies in finding a cultural connection within China’s rich and diverse traditions to make these views resonate. It is not merely about importing a Western perspective but about weaving it into the intricate fabric of Chinese beliefs and values. Otherwise, these Western interpretations of autism, rooted in brain science, risk providing mere solace for parents rather than fostering genuine understanding and acceptance of children with autism. In this context, one avenue China could consider is to develop psychoeducational programs for parents. Under the guidance of professionals, these programs can empower parents to understand the varied perspectives on autism etiology and equip them with the skills to explain autism and its associated symptoms to others in their daily lives [62].

### 5.2. Intergenerational Parenting

Previous studies have emphasized the significant stress caused by familial issues, especially when a child with autism challenges traditional family prestige and filial expectations [31]. However, our study showed that today’s parents of young children with autism felt less of a burden from these traditional views. This situation may be attributed to the recent phenomenon that traditional family values, known as “familism”, are descending in modern China. Specifically, as family structures become smaller and due to the long-term implementation of the one-child policy until 2016, the older generation is gradually diminishing its strong emphasis on traditional expectations of family prestige and reciprocal filial piety from descendants; instead, they are focusing more on the growth and well-being of their grandchildren [63]. In the meantime, we found that among the study participants, while mothers remained the primary caregivers for raising children with autism, few of them faced blame or isolation within their households due to having a child with autism. This finding sharply contrasts with previous research [32]. This departure should be attributed to the marital status of all study participants rather than cultural changes, as recent research indicates that traditional gender role expectations remain deeply entrenched in China [64].

An unexpected finding is that grandparents of the child with autism often resist acknowledging the diagnosis and are hesitant to embrace early interventions. While their active involvement in caregiving reflects the deep-rooted familial bonds inherent in Chinese culture [39], these differences in attitudes toward autism treatment contribute to increased parenting stress among the younger generation. Addressing this issue presents a complex task. On one hand, the practice of co-parenting has been deeply embedded due to collectivist values shared by both parents and grandparents [65]. On the other hand, persistent disagreements could have adverse effects on children’s development, such as the development of their effortful control [66]. Indeed, research on raising children with autism in Western countries has noted that Caucasian custodial grandparents often need to continuously adjust their roles and navigate space boundaries in caregiving within the family dynamics [67]. However, in China, the adaptations brought about by co-parenting warrant more attention. It not only necessitates family education and therapeutic approaches that bridge generational differences [68] but also demands sensitive handling due to the relational dependency and boundary complexities imposed by a collectivist culture.

### 5.3. Interactions with Professionals

In Chinese culture, parents often refrain from voicing different opinions, which is a behavior deeply rooted in longstanding social norms that emphasize reverence for authority [11,12,13,14,15,16,17,18,19,20,21,22,23]. Even well-educated parents in our study adhered to these traditional dynamics when interacting with professionals. This highlights the pervasive role of cultural norms over individual attributes or educational backgrounds.

The traditional practice of cultivating “guanxi” and “renqing” adds complexities to these dynamics. These conventions, which are historically crucial in Chinese interpersonal interactions, ensure reciprocity and balance in relationships [69]. Some parents feel that by gifting therapists and teachers, they can secure better professional support for their children, as reported in a recent study [70]. However, this approach is not without its challenges. Parents often feel anxious due to financial pressures and the behaviors of other parents. Some wonder whether they should match the generosity of other parents, while others worry about fairness for those who cannot afford such gifts. Consequently, the coping strategy clouds their perception of equity and social justice [71]. This issue urgently calls for government attention, since it is indicative of the unequal distribution of early intervention resources [72] or a lack of policies to regulate service provision. As a result, parents are compelled to use their own resources to secure the quality public services to which they are rightfully entitled.

However, despite these longstanding challenges, there are also glimmers of change emerging. The term “fight” used by a parent reflects a departure from the traditional passive stance and signifies a heightened awareness of rights within the special needs community. Furthermore, a mother’s efforts to integrate special education services into a mainstream preschool indicate the growing parental involvement in instigating change. Nevertheless, there is a notable absence of professional support for such advocacy [73]. Overall, whether parents choose to remain silent about their dissent, resort to giving gifts to professionals, exhibit assertive awareness of their rights, or actively seek changes, all these actions represent a plea: they need support to ensure their voices are heard.

### 5.4. Adjusting Parenting Styles

In line with the previous studies [39,40], the traditional Chinese parenting approach, characterized by its authoritarian nature, remains prevalent among parents. This authoritarian style emphasizes discipline as a primary means of addressing children’s behavioral challenges, often using tactics such as behavioral reinforcement and, at times, punishment [74]. Notably, parents of children with mild autism tend to show a distinct inclination toward an authoritative style, which is driven by their aspirations for their child’s improvement and successful social integration into society in the future [75].

However, evidence also points to a transformative shift in parenting approaches [76]. Parents are increasingly recognizing the tension and challenges introduced by an overly authoritarian approach, especially concerning their child’s emotional well-being. This introspection is driven by their realization of the conflicting roles they play as caregivers versus strict trainers. In particular, there is an indication found from our study that the adoption of applied behavior analysis (ABA) might further intensify these role conflicts, which is possibly because the intervention approach inherently promotes structured, consistent, and sometimes intensive behavior modification techniques [77]. When applied within the Chinese cultural context, where traditional parenting often leans toward authoritative methods, ABA could potentially push parents even further into the role of a trainer. This heightened emphasis on behavior modification might overshadow the nurturing aspect of parenting, exacerbating the tension between the dual roles of caregiver and enforcer. Furthermore, ABA’s structured methodologies may not always align with a child’s emotional needs, making it challenging for parents to adequately address these needs while adhering to the prescribed intervention strategies. All of these factors raise a pivotal issue about the applicability of ABA in the Chinese cultural context [78].

The dilemma of balancing discipline with emotional support highlights the limitations of using problem-focused strategies to cope with parenting stress within the Chinese cultural context. In their efforts to adjust their parenting practices, parents often neglect emotion-focused coping mechanisms that could alleviate their struggles, such as self-acceptance, self-compassion, and self-encouragement. This tendency may be a result of the high levels of self-denial and stoicism frequently displayed by Chinese parents [37]. Such tendencies further suggest that today’s parents of young children with autism have not yet fully detached from the influence of traditional culture, which often portrays parents in a self-sacrificial light. Therefore, alleviating parenting anxieties not only revolves around adopting more appropriate child-rearing methods but also entails redefining the relationship and dynamics between the caregiver and the child. Emphasizing self-affirmation and self-care does not equate to diminishing care for the child; on the contrary, it enhances it.

### 5.5. Challenges of Social Integration

In today’s Chinese society, a deeply ingrained stigma associated with mental illnesses remains prevalent, placing parents of children with autism in a challenging position where they often experience feelings of isolation and misunderstanding [25]. Compounding this challenge, some social phenomena in contemporary China have exacerbated this stigma. The media’s skewed portrayal of children with autism further amplifies these misconceptions, often depicting them as either exceptionally talented or extremely difficult [79]. Furthermore, emerging cultural tendencies, especially in urban areas such as Beijing, place a strong emphasis on competitive parenting and heightened expectations for early child development [29]. Such modern perspectives intensify the sense of exclusion experienced by these parents. These compounded stressors in an environment already rife with challenges may lead parents to adopt avoidance strategies, further hampering social integration. Thus, to effectively alleviate this parental stress, there needs to be a comprehensive reassessment of both entrenched stigmatization and the impact of these new cultural norms.

Nevertheless, amid these challenges, there are signs of change and resilience. Some parents are courageously stepping forward, sharing their experiences with a trusted circle, fostering understanding, and feeling a sense of belonging. These intimate interactions seem to have a more substantial impact on dispelling misconceptions than the mass media, which sometimes perpetuates stereotypes [79]. It is notable that these coping strategies and advocacy efforts can, in turn, reshape social perceptions. Government and non-governmental organizations can play pivotal roles in this regard. By empowering parents collectively, facilitating their voices, and fostering their initiatives, a more inclusive society can begin to take shape [80].

## 6. Study Limitations

In analyzing the results, this qualitative study highlighted the unique characteristics of parents raising children with autism in contemporary China. However, it is essential to note certain limitations. First, a significant proportion of our study participants were highly educated. Additionally, the two participants without a Bachelor’s degree were introduced to the study through referrals from highly educated parents. This educational homogeneity suggests that our findings might not be representative of all groups of parents, particularly those with lower educational and income backgrounds. Second, compared with smaller cities and villages, Beijing has a richer array of early intervention resources and superior access to information for families of children with autism. While this environment can accentuate the emerging cultural shifts, our findings may not be generalizable to other regions, especially rural settings where traditional cultural values and practices might exert more influence. Third, participants in our study were primarily recruited through professional referrals, which was complemented by snowball sampling. This recruitment method may introduce a selection bias, potentially excluding parents less likely to seek early intervention services due to traditional beliefs or concerns. Lastly, in our effort to preserve the richness of information related to cultural influences, we made a conscious decision not to exclude Participant 12, who was the sole participant reporting a low-income level. We also conducted a detailed analysis of this interviewee’s background and found that her economic needs were closely related to her role as a full-time mother, but she did not report that the income level exerted significant parenting stress. Nonetheless, we acknowledge that economic hardship can contribute significantly to parenting stress and coping experiences, as consistently documented in previous studies [18,19]. Future research endeavors need to delve deeper into the complex interaction between culture and income.

## 7. Conclusions

The experiences of Chinese parents raising children with autism are neither uniform nor static. Deeply rooted in traditional Chinese culture, their parenting practices are often influenced by longstanding cultural beliefs. However, it is crucial to recognize the dynamic and evolving nature of these experiences. Today’s generation of parents, shaped by both local traditions and global insights, are offering fresh perspectives. Positioned at the crossroads of tradition and modernity, they are uniquely capable of navigating and reshaping discussions on autism, intergenerational dynamics, early interventions, and social inclusion. Their approach is not a mere departure from the past but a fusion of tradition with contemporary understandings. Amid these interactions, there is a strong call for family-centered support. This support goes beyond just addressing immediate concerns—it amplifies the voices of these families in the broader community, fostering unity in their advocacy for their children’s well-being.

## Figures and Tables

**Table 1:** Study participants.

Participant #	Relationship	Birth Decade	Education	Self-Report Income	Child’s Sex	Child’s Age	Age at Diagnosis
1	Mother	1970s	College	Middle	Boy	6	3
2	Mother	1980s	Higher than college	Middle	Boy	4	2
3	Mother	1980s	Higher than college	Middle	Boy	8	3
4	Father	1980s	Doctorate	Middle	Boy	4	3
5	Mother	1980s	Master	Middle	Boy	6	2
6	Mother	1980s	Middle school	Middle	Girl	5	3
7	Father	1980s	Master	Middle	Girl	4	3
8	Mother	1980s	Master	Middle	Boy	6	4
9	Father	1980s	Doctorate	Middle	Boy	3	2
10	Mother	1980s	Master	Middle	Boy	5	2
11	Mother	1980s	Master	Middle	Boy	5	2
12	Mother	1980s	College	Low	Boy	5	3

## Data Availability

The data presented in this study are available upon request from the corresponding author. The data are not publicly available due to confidentiality and research ethics.

## Appendix A. Semi-Structured Interview Guide

Background Questions:

1. When were you born (e.g., born after the 1970s, 1980s, or 1990s)?
2. How many children do you have?
3. How old is/are your child/children with autism?
4. What is the gender of the child?
5. Where was the child born?
6. When did the child/children get an autism diagnosis?

Experience Questions:

1. How did you recognize the symptoms of autism in your child? Probing questions include:
  - What led you to seek a diagnosis?
  - Can you describe your journey toward accepting the diagnosis?

2. How did you initially decide to initiate treatment and intervention for your child? Probing questions include:
  - How did you gather information about available treatments and interventions?
  - What challenges did you encounter while searching for treatments and interventions?
  - What helped you find suitable treatments and interventions?

3. How did you choose treatment and intervention services for your child? Probing questions include:
  - What criteria influenced your choice of treatments and services?
  - How has the service you use changed over time?
  - Could you share your thoughts on your relationship with service providers?

4. How did you manage parenting your child at home? Probing questions include:
  - Did you implement interventions at home?
  - How did you, your spouse or parents co-parent your child?
  - Did you encounter any challenging parenting situations, and if so, how did you handle them?

5. What are your feelings regarding social inclusion for children with autism? Probing questions include:
  - Have you ever experienced marginalization due to your child’s diagnosis?
  - Can you recall any situations in public that caused you stress?

6. In your opinion, what cultural beliefs influence your experiences? Probing questions include:
  - Do you perceive any sigma towards autism or disabilities? Why or why not?
  - Have you ever felt reluctant to seek treatments and interventions due to cultural concerns? Why or why not?
  - Have you ever withheld your opinions to defer to authority figures and experts? Why or why not?
  - Any other cultural influence except those just mentioned?

Socioeconomic Background Questions:

1. What is your educational background?
  - If the interviewee appears uncomfortable with this question, instead ask, “Have you attended college?
2. How would you describe your family income level (e.g., high, middle, low)?
  - If the interviewee appears uncomfortable with this question, skip this question instead.

## Appendix B. Data Analysis Process

**Table A1:** Analysis steps from initial coding matrix to themes.

Initial Coding Matrix Derived from Deductive Analysis	Sub-Concepts Derived from Inductive Analysis	Synthesized Sub-Themes	Themes
1. Past sins belief in relation to autism [25] 2. Confusion in Chinese translation of autism [27] 3. Shame and “losing face” feelings [28] 4. Reframing as a coping strategy [37]	1. Western perspectives on autism	1. Traditional negative views on autism 2. Western neutral views on autism 3. Persistent confusion regarding autism’s nature	Blending tradition and modernity in perceptions of autism
1. Blame for family honor, disgrace [29] 2. Disruption of filial piety expectations [31,32] 3. Intergenerational co-parenting [39] 4. Family conflicts [39]	1. Generational attitudes toward autism and early intervention 2. Family boundary tensions	1. Reduced blame for giving birth to a child with autism 2. Tension arising from grandparents’ denial of autism 3. Adjusting boundaries with grandparents	Generational tension in parenting children with autism
1. Reluctance to seek professional help [4,34] 2. Deference to authority figures [34,35,36] 3. Harmony keeping through holding opinions [34,35,36]	1. Awareness of rights 2. Collaboration initiatives 3. “Renqing” investment to cultivate professional relationships *	1. Persistent obedience patterns with professionals 2. Stressed due to the costs of “renqing” investment 3. Asserting rights and initiating collaboration	Balancing unequal professional relationships
1. Emphasis on the roles of trainers and educators [37,38] 2. Problem-focused coping to address children’s needs [37,38] 3. Stress from role conflicts [40] 4. Self-sacrifice and self-control [40,41]	1. Balancing discipline and freedom	1. Dominance of authoritarian styles 2. Inclination toward nurturing love and care 3. Dilemma between reducing discipline and achieving progress	Adjusting authoritarian parenting styles
1. Feeling marginalized due to disability discrimination [28] 2. Hiding children with autism at home [33] 3. Avoiding social interactions [22]	1. Stress from competitive parenting 2. Stress from urban educational culture 3. Biased media portrayals 4. Expanding social circles	1. Persistent stigma-related stress 2. Contemporary environmental stress 3. Changes at the interpersonal level	Addressing the modern challenges of social marginalization

* Although this code is derived from an inductive coding process, it is considered a coping strategy in line with tradition, as discussed extensively in previous research related to Chinese culture [70,71].
